# Chaos in high-dimensional dissipative dynamical systems

**DOI:** 10.1038/srep12506

**Published:** 2015-07-30

**Authors:** Iaroslav Ispolatov, Vaibhav Madhok, Sebastian Allende, Michael Doebeli

**Affiliations:** 1 Departamento de Fisica, Universidad de Santiago de Chile, Santiago, Chile; 2 Department of Zoology and Department of Mathematics, University of British Columbia, Vancouver, BC V6T 1Z4 Canada

## Abstract

For dissipative dynamical systems described by a system of ordinary differential equations, we address the question of how the probability of chaotic dynamics increases with the dimensionality of the phase space. We find that for a system of *d* globally coupled ODE’s with quadratic and cubic non-linearities with randomly chosen coefficients and initial conditions, the probability of a trajectory to be chaotic increases universally from ~10^−5^ − 10^−4^ for *d* = 3 to essentially one for *d* ~ 50. In the limit of large *d*, the invariant measure of the dynamical systems exhibits universal scaling that depends on the degree of non-linearity, but not on the choice of coefficients, and the largest Lyapunov exponent converges to a universal scaling limit. Using statistical arguments, we provide analytical explanations for the observed scaling, universality, and for the probability of chaos.

In many standard texts, a transition from classical deterministic description to statistical physics is justified by the prevalence of chaotic and ergodic behavior as more degrees of freedom are considered. However, quantitative details of such transitions to chaos in dynamical systems apparently remain elusive. Most frequently, the connection between system dimension and the probability of chaos has been studied in high-dimensional discrete maps^1^,^2^,^3^,^4^,^5^,^6^. In continuous time, substantial work has been done with Hamiltonian systems, and the mechanisms by which integrable Hamiltonian systems become chaotic as the strength of non-linearity is increased are now fairly well understood^7^. The related issue of the “extensivity” of the Kolmogorov-Sinai entropy was investigated in^8^, and the dependence of the probability of chaos in a chain of locally coupled harmonic oscillators on its length has been studied in^9^. In dissipative systems, the low dimensional chaos has been extensively explored, mostly numerically (e.g.^10^). Sprott and co-workers have studied the prevalence and degree of chaos in high-dimensional networks described by a system of globally coupled ordinary differential equations with a hyperbolic tangent non-linearity. They have shown the prevalence of chaotic trajectories in high-dimensional systems with sufficiently weak damping^11^,^12^,^13^. However, a systematic investigation of the probability of chaos as a function of the dimension of phase space in dissipative, continuos-time dynamical systems with simple and generic non-linearities does not seem to be available.

Here we present the results of our attempt to perform such an investigation. In the following we show that the probability that the solution of a generic *d*-dimensional system of ODEs with quadratic and cubic non-linearities (1,2,3) is chaotic universally increases from ~10^−4^ − 10^−5^ for *d* = 3 to essentially 1 for large *d*. The results of our numerical investigations are then explained analytically, using a combination of scaling and statistical methods. These results are an extension and generalization of an investigation of the prevalence of chaos in the dynamics of high-dimensional phenotypes under frequency-dependent natural selection^14^. However, the applicability and significance of our results is not limited to biological evolution, and in principle extends to dynamical systems in statistical and nonlinear physics, hydrodynamics, plasma physics, control theory, and social and economic studies.

To investigate the statistics of trajectories, we numerically solve the following systems of equations which contain second- and third-order nonlinear terms of a general form,


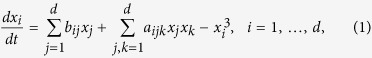



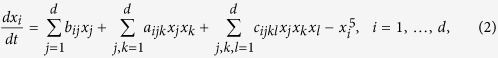



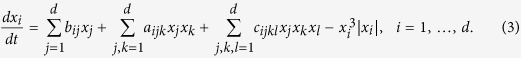


The coefficients {*a*}, {*b*}, and {*c*} were randomly and independently drawn from Gaussian distributions with zero mean and unit variance. The highest-order terms, 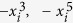, and 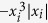 were introduced to ensure confinement of all trajectories to a finite volume of phase space, thus excluding divergent scenarios. Since the probability of randomly picking coefficients {*a*}, {*b*}, and {*c*} corresponding to a Hamiltonian system is zero, we refer to systems of the form (1,2,3) as generally dissipative. In^14^, we integrated system (1) for each dimension *d* using a 4th-order Runge-Kutta method for 50 sets of the coefficients *b*_*ij*_ and *a*_*ijk*_, each with 4 sets of random initial conditions. This procedure was repeated for (1) in the current work. Numerical solution of (2) and (3) is more complex and computationally extensive. We integrated systems (2) and (3) using a 5th-order Runge-Kutta adaptive step method for 100 (50 for *d* ≥ 30) sets of the coefficients *b*_*ij*_, *a*_*ijk*_, and *c*_*ijkl*_ for each dimension, but starting from just a single random choice of initial conditions. For each trajectory we determined the Largest Lyapunov Exponent (LLE) by perturbing the trajectory by a small magnitude *δx*_0_ in a random direction, integrating both trajectories in parallel for time *τ*, measuring the distance between trajectories *δx*_*τ*_, rescaling the separation between trajectories back to *δx*_0_, and continuing this for the course of the simulation. The LLE was calculated as


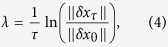


and subsequently averaged over the trajectory. The time of integration was chosen such that the average LLE saturated to a constant value and it was usually not less than ~10^4^/*d*^*β*^ with *β* = 2, 3, 9/2 for Eq. (1, 2, 3). We explain this scaling below. In cases when a trajectory converged to a stable fixed point and the LLE was persistently negative, the integration was stopped.

By visually inspecting the trajectories that have not converged to a fixed point, we derived the following classification: The trajectories with *λ* ~ *d*^*β*^ (with the proportionality coefficient being of the order of 0.1) are chaotic, while the trajectories with *λ* ~ 1 are “quasiperiodic”, i.e. converging to a limit cycle. In the latter case, the deviation of the average value of the LLE from *λ* = 0, which is the expected value for a periodic attractor, is attributed to transient chaos^15^, which is characterized by positive LLE’s. Hence in such cases, the average LLE contained a positive contribution from the transitory chaos and *λ* = 0 from the time spent on the quasi-periodic attractor, making the average much less than *d*^*β*^. The rare intermediate cases of LLE between *λ* ~ *d*^*β*^ and *λ* ~ 1 were inspected and classified individually. For Eq. (1), for which the simulations were less computationally-expensive, we implemented a more refined method of measuring *λ*, first allowing considerable time for the system to settle on the attractor and only then starting to average *λ*. Thus we were able to narrow the range of LLE for the quasiperiodic trajectories to |*λ*| ≤ 0.1, and considered all trajectories with *λ* ≥ 0.1 to be chaotic. In any case, the precise distinction between quasiperiodic and chaotic trajectories is not important for the main conclusion of our paper, as the fraction of quasiperiodic trajectories never exceeds 25% and vanishes for higher dimensions.

Our main result is that for all considered types of non-linearity the probability of chaos increases with the dimension of the phase space, Fig. 1. In particular, the numerical simulations for (1,2,3) suggest that, essentially all trajectories become chaotic for 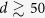. Our simulations also indicate that already for intermediate dimensions 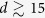, the majority of chaotic trajectories essentially fill out the available phase space, i.e., become ergodic (Fig. 2, left panel). In such a regime the probability density *P*(*x*_*i*_) for each coordinate of the chaotic attractor approaches a universal scaling form that depends neither on the choice of coefficients {*a*}, {*b*}, {*c*} nor on the dimension *d*, Fig. 2. Furthermore, the LLEs also exhibits apparent scaling behavior, Fig. 3.

Below we explain the scaling and statistical properties of the large-*d* limit of (1,2,3) First, consider the scaling of the spatial coordinates *x*_*i*_ ~ *d*^*α*^ and the LLEs *λ* ~ *d*^*β*^, illustrated in Figs 2 and 3. Consider the general case of a dynamical system similar to (1,2,3) with the *n*th-order highest nonlinear term a the |*x*_*i*_|^*m*^sgn(*x*_*i*_), *m* > *n* diagonal confining term,


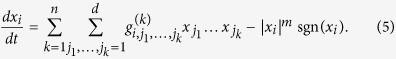


Since the coefficients 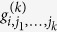 in (5) are drawn randomly, it is reasonable to assume that each coordinate has a similar scale, *x*_*i*_ ~ *x* and (5) becomes


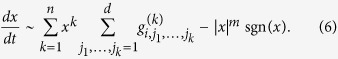


Here the 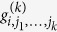 are identically distributed random terms with zero mean and unit variance, and a typical value of the sum of *d*^*k*^ such terms is the standard deviation 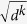, which yields the following scaling relation:


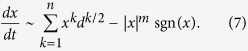


Introducing new variables,


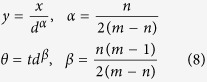


we convert (7) into


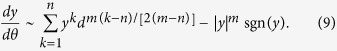


On the right-hand side of (9), the highest-order *k* = *n* term and the |*y*|^*m*^sgn(*y*) term do not depend on *d* while the lower-order terms with *k* < *n* vanish in the limit of *d* ≫ 1. The transformation (8) explains the observed scaling of the size of chaotic attractors and the LLEs (whose dimension is the inverse of time) shown in Figs 2 and 3. A more detailed example of the above derivation for Eq. (1) is given in^14^. To explain the shape of the universal probability density *P*(*y*) shown in Fig. 2, we ignore the irrelevant low-order terms and replace the leading nonlinear *n*th-order term (quadratic in (1) and cubic in (2,3)) by a stochastic function *f*(*θ*). This is done observing that for large *d*, the majority of the terms comprising the 
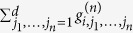
 do not contain *x*_*i*_ and can be approximated as independent random variables. Since 〈*g*^2^〉 = 1 by definition, it follows from the Central Limit theorem that this sum is a Gaussian random variable with variance *D* = *d*^*n*^〈*x*^2^〉^*n*^. This leads to the following approximation of (6) in the rescaled variables *y* and *θ* of (8):


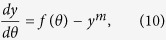


where *f*(*θ*) is Gaussian process with dispersion *D*. To calculate the invariant measure of this process we approximate *f*(*t*) by a jump process which takes constant Gaussian-distributed values *f*_*i*_ during time intervals drawn from a uniform distribution with an average period *τ*. We solve to the scaling equation (10) self-consistently, computing 〈*y*^2^〉^*n*^ from the histogram of the trajectory *y*(*θ*) produced via (10). Varying *τ*, we find the best fit to the observed *P*(*y*), which is shown as dashed lines in Fig. 2. Given the approximate nature of the temporal behaviour of *f*(*θ*) the fit seems quite satisfactory and yields *τ*^(1)^ = 3.85 for (1), *τ*^(2)^ = 5.64 for (2), and *τ*^(3)^ = 6.63 for (3) Note that the estimate for *τ*^(1)^ = 3.85 is not very different from the large-*d* asymptotic value of the corresponding rescaled LLE 1/λ^*^ ≈ 4.26, (see Fig. 3), which characterizes the typical correlation time of the system. The lack of data points for very high dimensions for systems (2) and (3) makes the extrapolation of the corresponding curves in Fig. 3 ambiguous, but the plotted values of 1/*λ* are of the same order of magnitude as the corresponding correlation times *τ*.

Next we provide a statistical explanation for the probability of chaos as a function of the dimension *d*, as illustrated in Fig. 1. As the fraction of quasiperiodic trajectories becomes negligible in high dimensions, in the following we refer to all non-stationary attractors as chaotic. Consider stationary points of the dynamical systems (1,2,3). Since a system of *d m*th-order algebraic equations generally has *md* solutions (sometime coinciding), the dynamical system (1) has *md* stationary points x^*^. For our derivation, we assume that the system is chaotic or quasiperiodic if all these stationary points are unstable in at least one direction, i.e., if at each stationary point x^*^ at least one eigenvalue of the local Jacobian matrix *J*(x^*^) has a positive real part. We assume that for sufficiently high *d*, all Jacobian eigenvalues are statistically independent. This assumption of weakening correlations between dimensions as the number of dimensions increase is a rather strong approximation without which it seems impossible to derive analytical estimates, and which seems to result in reasonable results (see below). Denoting the probability that the real part of an eigenvalue is negative by *P*_*neg*_, the probability that at least one out of *d* eigenvalues of the Jacobian at a stationary point has a positive real part is 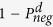. Hence the probability of chaos is


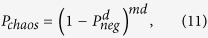


indicating that for any *P*_*neg*_ = 1 − *ε* < 1, the system becomes predominantly chaotic for 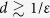. Specifically, consider the example of system (2) with cubic non-linearities. If x^*^ is a stationary point of (2), the elements of the Jacobian matrix 
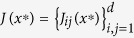
 consist of two terms,


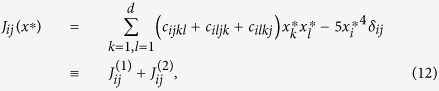


where {*δ*_*ij*_} is the identity matrix. As above, we ignored low-order terms present in (5), because such terms are irrelevant for large *d*. We assume that the distribution of 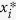 is the same as for the coordinates *x*_*i*_ themselves and is given by the universal invariant measure shown in Fig. 2. We also consider the two terms 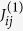 and 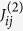 as statistically independent. The first term, 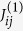, is a sum of 3*d*^2^ ≫ 1 random variables with zero mean and a finite variance. Taking into account that the dispersions of *c*_*ijkl*_ are one, and *x*_*i*_ and {*c*_*ijkl*_} are uncorrelated (this follows from the observed independence of *P*(*x*) and the choice of {*c*}) the Central Limit theorem states that this sum is a Gaussian-distributed variable with zero mean and dispersion *σ*^2^ = 3*d*^2^〈*x*^2^〉^2^. It follows from “Girko’s circular law”^16^,^17^ that eigenvalues of a random *d* × *d*-matrix with Gaussian-distributed elements with zero mean and unit variance are uniformly distributed on a disk in the complex plane with radius 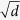. Thus, the eigenvalues of 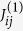 are uniformly distributed on a disk with radius 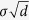. The probability for an eigenvalue of 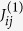 to have real part 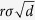, with |*r*| ≤ 1, is then proportional to the length of the chord intersecting the radius of the disk at the point *r*,


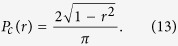


(The factor 2/*π* normalizes *P*_*c*_(*r*) to one.) The probability distribution of the second, diagonal, term of the Jacobian, 
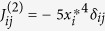
 is defined by the invariant measure *P*(*y*), given by (10) and shown in Fig. 2. It follows from scaling (8) that both 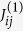 and 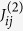 contribute terms of order *d*^3^ to the eigenvalues of the Jacobian. The contribution from 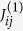 may have a positive or a negative real part with equal probability 1/2. The contribution from 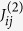 is always negative and has magnitude 5*y*^4^ with probability *P*(*y*). It follows that the probability that the sum of the two contributions has negative real part is


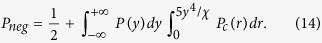


where 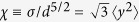. Integration on *dr* produces


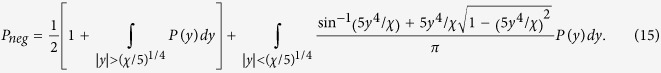


Using the numerical data for *P*(*y*) shown in Fig. 2 we calculate *χ* ≈ 0.446 and perform numerical integration of *P*(*y*) to obtain 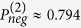. A similar analysis for Eqs (1) (14 and (3) yields 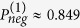 and 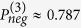, respectively. Substituting these values into Eq. (11) provides a reasonable fit for the observed probability of chaos, as illustrated by the dashed lines in 1. An increasing discrepancy for lower *d* could be attributed to the facts that the systems reach truly scaling regime for *d* → ∞ and the histograms in Fig. 2 are measured for rather high *d* ≥ 45.

To summarize, we have presented numerical evidence that the behaviour of deterministic dissipative dynamical systems in continuous time universally becomes chaotic and ergodic as the dimension of the phase space becomes large (*d* ~ 50 in the three cases we studied). Interestingly, a similar threshold was observed for the transition to ubiquity of chaos in a networks with *tanh*(*x*) non-linearity^11^,^12^,^13^. We note that the quadratic and cubic non-linearities considered here can be interpreted as the first few non-linear terms in the expansion of more complex non-linear dynamical systems, possibly extending the applicability of our results. We have also provided analytical explanations for the observed ubiquity of chaos and for the universality of the density distribution of chaotic trajectories. The similarity of the three panels in Fig. 1 and the apparently general applicability of Eq. (11) suggest that the observed transition to chaos is not limited to the three cases considered here and instead universally occurs in all high-dimensional nonlinear dissipative dynamical systems. Our observations may also provide important insights into chaotic behavior of continuous systems described by partial differential equations, which are often digitized as systems of many ordinary differential equations. Furthermore, our results are directly applicable and important not only to pure nonlinear physics, but also to other fields where non-linear interactions are common, such as hydrodynamics and plasma physics, optics, systems biology, evolution, and control theory.

One of the goals of this work was to illustrate the transition to chaos and ergodicity in high-dimensional phase space, a frequently used yet rarely precisely stated argument in the formal justification of statistical mechanics. Nevertheless, to explain our results we use scaling and probabilistic arguments borrowed from statistical physics. Thus, our results are an attempt to use statistical physics to establish a basic “phase diagram” of dynamical systems.

## Additional Information

**How to cite this article**: Ispolatov, I. *et al*. Chaos in high-dimensional dissipative dynamical systems. *Sci. Rep*. **5**, 12506; doi: 10.1038/srep12506 (2015).

## Figures and Tables

**Figure 1:**
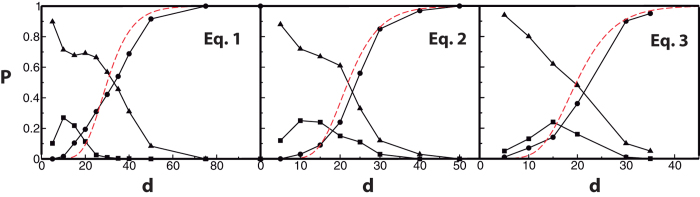
Numerically measured probability of different types of dynamics as a function of dimension *d* of the phase space for Eq. (1) (left panel), Eq. (2) (central panel), Eq. (3) (right panel): **•** - chaotic trajectories, ■ - limit cycles, ▲ - stable fixed points. For each case, the theoretical estimate for the probability of chaotic trajectories (see main text) is shown by a dashed line (red color online).

**Figure 2:**
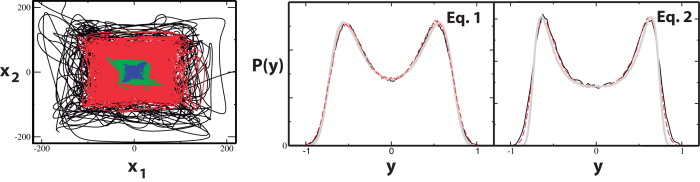
Scaling of the size of chaotic trajectories. Left panel: Examples of *x*_1_, *x*_2_ projections of trajectories for the dynamics described by (3) for *d* = 10 (blue), *d* = 15 (green), *d* = 30 (red), and *d* = 45(black), illustrating the scaling *x*_*i*_ ~ *d*^3/2^. Central panel: The probability density for the scaled coordinate *P*(*y*) vs. *y* = *x*/*d*^*α*^, *α* = 1 of the solution of Eq. (1) for *d* = 150 (solid black line), *d* = 100 (dashed red line), and the histogram of the solution of (10) (thick grey line). Right panel: The probability density for the scaled coordinate *P*(*y*) vs. *y* = *x*/*d*^*α*^, *α* = 3/4 of the solution of Eq. (2) for *d* = 65 (solid black line), *d* = 50 (dashed red line), and the histogram of the solution of (10) (thick grey line).

**Figure 3:**
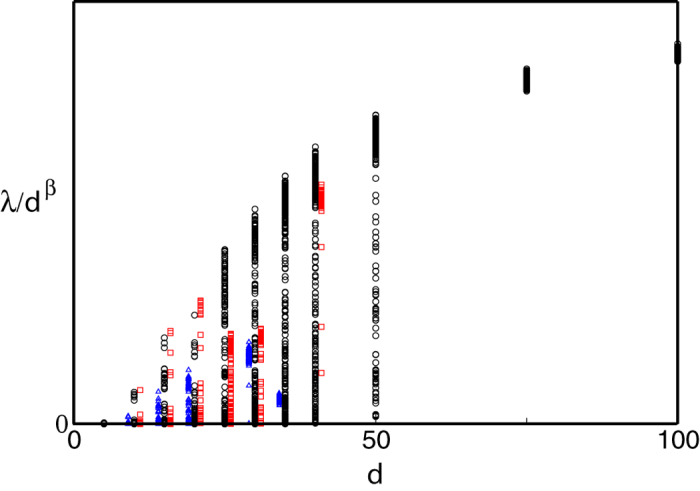
The scaled LLE *λ*/*d*^*β*^ as a function of the dimension *d* of phase space for (1); *β* = 2, black circle; (2), *β* = 3, square, shifted to the right, red online; and (3), triangle, shifted to the left, blue online, *β* = 9/2. For large *d*, the LLE for (1) extrapolates to *λ*/*d*^*β*^ → λ^*^ ≈ 0.235. (see main text).
